# Rare Dual Genetic Diagnosis of Wiskott-Aldrich Syndrome and Ghoshal Hematodiaphyseal Dysplasia: Clinical, Diagnostic, and Management Challenges

**DOI:** 10.7759/cureus.99572

**Published:** 2025-12-18

**Authors:** Eby P Baby, Shruti Verma, Abhishek Bhagel, Savitri Singh, Nita Radhakrishnan

**Affiliations:** 1 Department of Pediatric Hematology Oncology, Post Graduate Institute of Child Health, Noida, IND; 2 Department of Pathology, Post Graduate Institute of Child Health, Noida, IND

**Keywords:** bone marrow fibrosis, dual mutation, ghoshal hematodiaphyseal dysplasia, immunodeficiency, inherited anemia, wiskott-aldrich syndrome

## Abstract

Wiskott-Aldrich syndrome (WAS) is a rare X-linked immunodeficiency characterized by microthrombocytopenia, recurrent infections, eczema, and risk of autoimmunity or malignancy. Ghoshal hematodiaphyseal dysplasia (GHD) is an extremely rare autosomal recessive disorder caused by pathogenic variants in the *TBXAS1 *gene, leading to bone marrow fibrosis, transfusion-dependent anemia, and skeletal dysplasia. While each disorder individually is rare, their co-inheritance in the same patient has not been reported. With the increasing use of next-generation sequencing, dual genetic diagnoses are being recognized, particularly in consanguineous populations, and often present with blended phenotypes that complicate diagnosis and management. We describe a 14-month-old boy, the third child of consanguineous parents, presenting with transfusion-dependent anemia from early infancy, severe thrombocytopenia with microplatelets, recurrent bacterial infections, mild splenomegaly, and evidence of early marrow fibrosis. Genetic testing revealed a hemizygous frameshift mutation in the *WAS* gene (c.511del; p.Arg171GlufsTer90) consistent with WAS, along with a homozygous missense mutation in *TBXAS1 *(c.1235G>A; p.Arg412Gln), confirming GHD. Both variants were classified as pathogenic. The co-inheritance necessitated modification of treatment for both individual diseases. Supportive care with transfusions and antimicrobials was provided. This case highlights the first and exceptional occurrence of two rare inherited disorders: WAS and GHD in the same child. The blended phenotype, with early marrow fibrosis, could not be explained by either condition alone. Such dual diagnoses underscore the critical role of molecular testing in atypical pediatric cytopenias.

## Introduction

Inherited platelet disorders represent a clinically and genetically diverse group of conditions, many of which manifest in infancy or early childhood with cytopenias, recurrent infections, and bleeding diathesis [1]. Wiskott-Aldrich syndrome (WAS) and Ghoshal hematodiaphyseal dysplasia (GHD) are clinically distinct entities that can present with thrombocytopenia [2].

WAS is an X-linked recessive immunodeficiency disorder caused by mutations in the *WAS *gene located at Xp11.23. The incidence is estimated at 1-10 per million live male births worldwide [1]. The classical triad includes microthrombocytopenia, recurrent infections, and eczema, though autoimmune manifestations and malignancy are also recognized complications. Advances in molecular diagnostics have expanded the phenotypic spectrum, with some patients presenting with isolated thrombocytopenia or atypical immunodeficiency [1,3]. Hematopoietic stem cell transplantation (HSCT) remains the standard curative therapy, while gene therapy trials are underway [4].

In contrast, GHD is an autosomal recessive disorder caused by mutations in the *TBXAS1 *gene, which encodes thromboxane synthase. This enzyme is involved in the arachidonic acid pathway, and its deficiency leads to impaired hematopoiesis and progressive marrow fibrosis. Since its first description in 1988, fewer than 40 cases have been reported globally, making it one of the rarest inherited bone marrow failure syndromes [5,6]. Clinical features include transfusion-dependent anemia, thrombocytopenia, marrow fibrosis, and skeletal abnormalities such as diaphyseal expansion and cortical thickening [7]. Because of its extreme rarity, GHD is often overlooked, and diagnosis is typically achieved only through next-generation sequencing.

The co-existence of two distinct pathogenic mutations within the same child is exceedingly uncommon, but it becomes more plausible in populations with consanguinity or high rates of recessive disorders. Statistically, the chance of inheriting two independent rare disorders is the product of their individual incidences, which for WAS (~1-10 per million male individuals) [1] and GHD (<1 in 10 million globally, given <40 cases reported to date) [6] yields a combined probability that is astronomically small. Nevertheless, with the increasing use of broad genetic panels and exome sequencing, cases of dual molecular diagnoses are being increasingly recognized in pediatric genetics. Such blended phenotypes can obscure the clinical picture, delay diagnosis, and complicate management.

To date, there has been no prior report of co-inheritance of WAS and GHD. Here, we present a case of a 14-month-old boy with transfusion-dependent anemia, microthrombocytopenia, recurrent infections, and bone marrow fibrosis, found to harbor pathogenic variants in both *WAS *and *TBXAS1*. This report expands the phenotypic spectrum of inherited cytopenias and highlights the importance of molecular diagnostics, particularly in consanguineous families where the probability of dual pathogenic variants is higher than in the general population.

## Case presentation

The index patient was a 14-month-old boy, the third child of a consanguineous marriage. The family history was notable for one sibling who had died in infancy with similar complaints. His seven-year-old sister was alive and healthy with no clinical manifestations. He had another elder brother who was five years old and was also investigated for pallor and bruising. However, this boy was not evaluated further and was transfusion-free.

The index child had been symptomatic from early infancy. Easy bruising was first observed at the age of one month. From six months of age onwards, he experienced recurrent infections, including otitis media, pneumonia, and dysentery, which required multiple hospitalizations. At the same time, he also developed progressive anemia necessitating red blood cell transfusions approximately once every three to four months. Additional bleeding manifestations included an episode of epistaxis and one episode of gastrointestinal bleeding. On physical examination at 14 months of age, he was pale and was noted to have a prominent forehead, which was thought then to be “hemolytic facies.” Multiple bruises were evident on the skin, and the spleen was enlarged (1 cm along its axis). There was no hepatomegaly, and no eczematous lesions were observed. 

Laboratory investigations demonstrated bicytopenia. The complete blood count revealed a hemoglobin level of 8.97 g/dL (transfused a week prior), a white cell count of 12.3 × 10^9^/L with a normal differential, and a platelet count of 81.2 × 10^9^/L. The mean platelet volume was markedly reduced at 4.1 fL, consistent with microthrombocytopenia. With these findings, the possibility of inherited thrombocytopenia, such as WAS, was considered. However, severe anemia with recurrent transfusion requirement was unusual for WAS. He was evaluated to rule out autoimmune hemolytic anemia, which is often noted with WAS. The reticulocyte count was inappropriately low; corrected reticulocyte count was 0.1% suggesting impaired marrow function. The hemogram findings are compiled in Table 1. Peripheral smear examination revealed microthrombocytes, anisopoikilocytosis, nucleated red blood cells, and teardrop cells, findings that were unusual for isolated WAS. Direct Coomb’s test was negative.

**Table 1:** Complete hemogram MCV: mean corpuscular volume; MCH: mean corpuscular hemoglobin; MCHC: mean corpuscular hemoglobin concentration; MPV: mean platelet volume; RDW: red cell distribution width; WBC: white blood cell

Parameter	Patient’s value	Normal value (children)
Hemoglobin	8.97 g/dL	11.5-15.5 g/dL
Red cell count	4.02 million/µL	4.1-5.5 million/µL
MCV	71.2 fL	76-90 fL
MCH	22.3 pg	25-33 pg
MCHC	31.3 g/dL	32-36 g/dL
WBC count	12,300/µL	4,500-11,000/µL
Absolute neutrophil count	4,182/µL	1,500-8,000/µL
Platelet count	81,200/µL	150,000-450,000/µL
MPV	4.1 fL	7.5-11.5 fL
RDW	16.2%	11.5-14.5%
Corrected reticulocyte count	0.1%	0.5-1.5%

**Figure 1:**
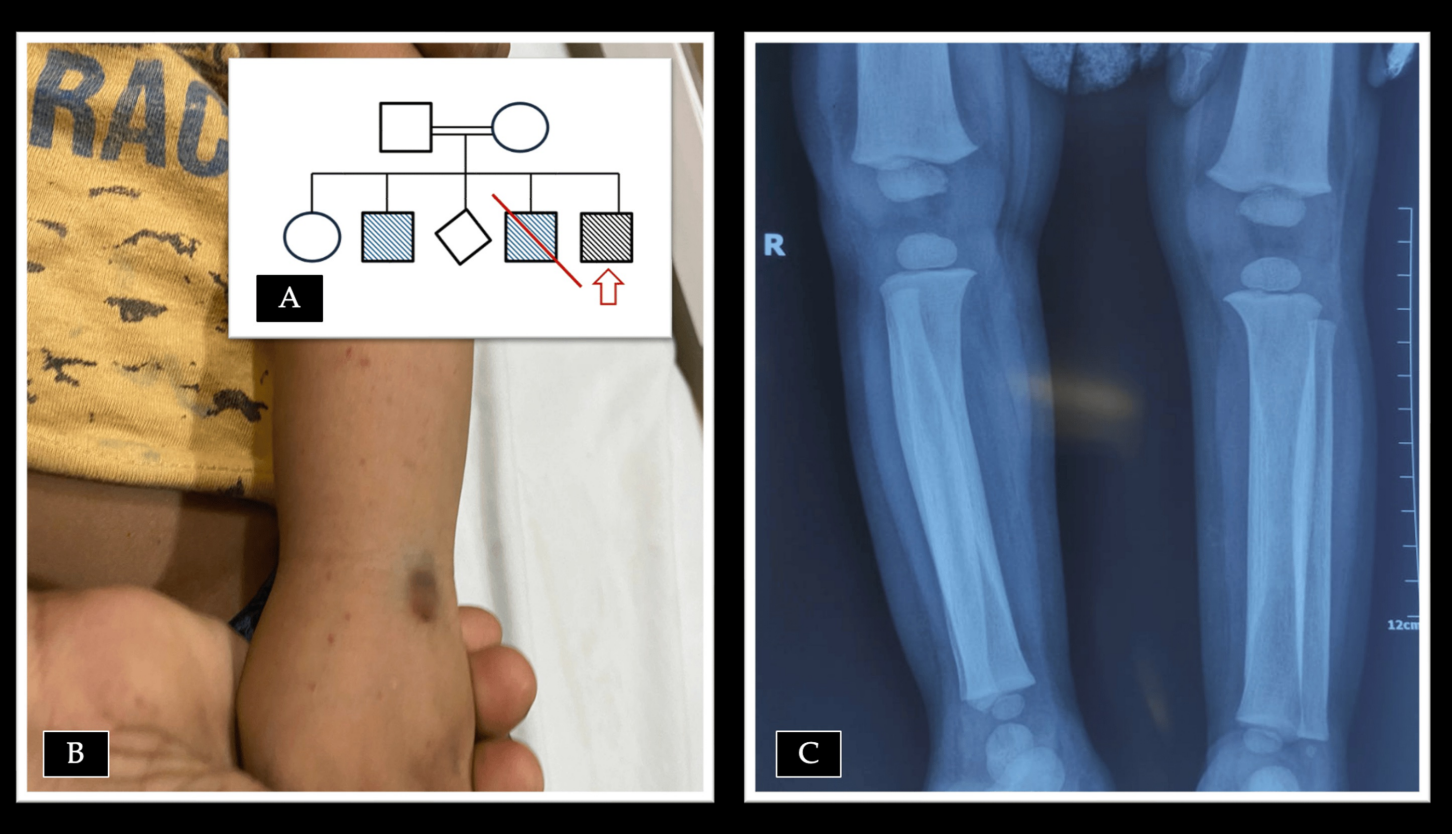
(A) Pedigree demonstrating consanguinity and multiple affected male siblings with early childhood deaths, suggestive of an inherited disorder. The red arrow indicates the index case. (B) Clinical photograph of the index child showing petechiae and ecchymosis over the left upper limb, reflecting thrombocytopenia consistent with Wiskott-Aldrich syndrome. (C) Radiograph of the long bones showing characteristic findings of Ghoshal hematodiaphyseal dysplasia, including symmetrical cortical thickening and fusiform diaphyseal widening with metaphyseal sparing.

**Figure 2:**
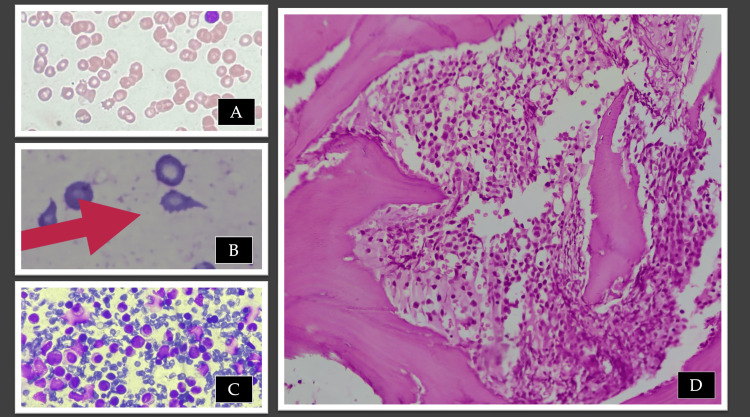
Hematopathological evaluation. (A) Peripheral blood smear (40×) showing microthrombocytopenia. Platelets are markedly reduced in number and appear abnormally small. (B) Peripheral blood smear (100×). Red cells show anisopoikilocytosis with occasional teardrop forms, suggesting associated marrow fibrosis. The red arrow indicates a teardrop-shaped red cell. (C) Bone marrow aspirate showing hypercellularity. (D) Bone marrow biopsy showing paratrabecular streaming and distorted architecture.

Bone marrow aspiration yielded scant cellular material, and the trephine biopsy demonstrated a hypercellular marrow with trilineage hematopoiesis and marked paratrabecular streaming, indicative of early myelofibrosis. Megakaryocytes were reduced in number and displayed abnormal morphology. These features raised the suspicion of a bone marrow failure syndrome or myelofibrosis. The pedigree chart and clinical photographs are illustrated in Figure 1, while the peripheral smear and bone marrow findings of the index patient are shown in Figure 2.

To clarify the diagnosis, next-generation sequencing was performed using whole-exome sequencing with variant search for inherited bone marrow failure syndromes. This analysis identified two pathogenic variants. The first was a hemizygous frameshift deletion in exon 6 of the *WAS *gene (c.511del; p.Arg171GlufsTer90), predicted to result in premature truncation of the protein and consistent with classical WAS. The second was a homozygous missense mutation in exon 11 of the *TBXAS1 *gene (c.1235G>A; p.Arg412Gln), a variant previously reported to be pathogenic and associated with GHD. Both the *WAS *frameshift mutation (c.511del; p.Arg171GlufsTer90) and the *TBXAS1 *missense mutation (c.1235G>A; p.Arg412Gln) were classified as pathogenic according to American College of Medical Genetics and Genomics (ACMG)/Association for Molecular Pathology (AMP) guidelines [8], supported by in silico prediction models, population databases, and prior literature reports of disease association. Radiograph of the long bones of the leg demonstrated symmetrical cortical thickening and fusiform expansion of the diaphyses with increased radiodensity.

Challenges in managing dual inheritance

The management of WAS has evolved considerably over recent decades, with HSCT now established as the treatment of choice and the only curative option. Early HSCT, particularly from a human leukocyte antigen (HLA)-matched sibling donor, is associated with survival rates exceeding 90% [4]. Unrelated donor and haploidentical transplantation outcomes have also improved with reduced-toxicity conditioning regimens and advanced graft manipulation techniques. Supportive management includes prophylactic antimicrobials, immunoglobulin replacement, and platelet transfusions for bleeding episodes.

The treatment of GHD remains challenging owing to its extreme rarity. Most children present with transfusion-dependent anemia, making red blood cell transfusions and infection control the cornerstone of supportive management. A substantial proportion of reported patients show hematologic improvement with low-dose corticosteroids, which can reduce transfusion requirements, as documented across multiple case reports and small series. More recently, targeted approaches that modulate the arachidonic acid-thromboxane pathway, including aspirin and non-steroidal anti-inflammatory drugs (NSAIDs), have been trialed, with case reports describing hematologic benefit in selected patients [7,9,10]. HSCT has not been widely reported in indexed literature, and current published evidence supports supportive therapy, corticosteroids, and emerging targeted therapy as the mainstay of management for this ultra-rare disorder.

The child was managed with supportive care. He received regular packed red blood cell transfusions to maintain his hemoglobin level above 9 g/dL. Prophylactic cotrimoxazole was prescribed, and intravenous immunoglobulin was administered during episodes of severe infection. Platelet transfusions were given in the setting of bleeding. In our patient, neither corticosteroids nor NSAIDs were a treatment option as they could potentially aggravate the complications, such as infections and bleeding, seen in thrombocytopenia. In view of WAS, hematopoietic stem cell transplantation was considered; however, the healthy sibling was not an HLA match. He was followed up for a year with transfusion support, following which he succumbed to pneumonia and pulmonary hemorrhage.

## Discussion

The co-inheritance of two inherited genetic disorders in a single individual, though rare, has growing recognition in clinical genetics, especially as whole exome/genome sequencing becomes more accessible. Cases with “blended phenotypes” can result when pathogenic variants in two different genes cause overlapping or interacting clinical effects. Such dual diagnoses complicate both diagnosis and management because the phenotype may deviate from what is expected of either disease alone.

In our patient, classical features of WAS with microthrombocytopenia, immunodeficiency, bleeding, and features more typical of GHD, such as transfusion-dependent anemia, marrow fibrosis, and skeletal findings, coexisted, thus compounding the disease severity. Individually, WAS does not usually cause marrow fibrosis or the severity of anemia observed; conversely, GHD does not cause the immune defects characteristic of WAS. The presence of both sets of features in the same child produced a blended phenotype that obscured the diagnosis: initial suspicion rested on WAS, but atypical findings (low reticulocyte count, teardrop cells, early fibrosis) prompted further genetic investigation that revealed the *TBXAS1 *mutation of GHD in addition to the known *WAS *gene defect.

Clinical spectrum and management of GHD

GHD typically manifests in early childhood with anemia, thrombocytopenia, and skeletal changes, including cortical thickening and diaphyseal expansion. The “hemolytic facies” noted in the patient, characterized by a prominent forehead and cheeks, was most likely attributable to the skeletal changes associated with GHD due to diaphyseal expansion. Bone marrow examination reveals progressive fibrosis, leading to transfusion dependence. Management is largely supportive, with blood transfusions, treatment of infections, and careful monitoring of bone health. Corticosteroids and aspirin have been employed in some cases to reduce marrow fibrosis and improve hematological parameters, given their modulatory effects on prostaglandin and thromboxane pathways. However, such therapies are not universally effective and remain palliative [9-11].

Management challenges in dual diagnosis

The co-existence of WAS introduced further complexity. In classical GHD, corticosteroids or aspirin may be considered as supportive therapy. In this child, both options were unsuitable. Corticosteroids are relatively contraindicated due to the underlying immunodeficiency of WAS, which would be exacerbated by steroid-induced immunosuppression. Aspirin, although potentially beneficial in GHD for modulating thromboxane pathways, is contraindicated in a child with severe thrombocytopenia and bleeding diathesis due to WAS, as it would significantly increase the risk of hemorrhage. In our case, identifying both mutations allowed for more precise prognostication and better family counseling for recurrence risk.

Reports of patients harboring two distinct genetic diseases, so-called dual genetic diagnoses or blended phenotypes, are increasingly recognized with the widespread use of exome and genome sequencing. Large cohort studies suggest that although uncommon, the phenomenon is not negligible: in a diagnostic exome cohort of 7,698 individuals, 33 patients (0.43%) were found to have two definitive genetic diagnoses [11-13]. Correia-Costa et al. reviewed 106 individuals with dual autosomal recessive disorders reported in the literature, further underscoring that such blended phenotypes, while rare, are a clinically relevant entity [14]. Combining an X-linked disorder such as WAS with an autosomal recessive condition like GHD is exceptionally rare [14-16].

We wish to highlight here that bone marrow fibrosis in children is rare and usually reflects an underlying congenital, metabolic, or secondary process rather than the myeloproliferative neoplasms seen in adults. Infections (tuberculosis), malignancies (Hodgkin lymphoma, leukemias, neuroblastoma, etc.), vitamin D deficiency, and storage disorders like Gaucher disease can produce secondary fibrosis due to infiltration and altered bone remodeling. Idiopathic myelofibrosis of infancy and childhood is extremely rare and is considered only after other causes are excluded [17]. GHD should be suspected when marrow fibrosis occurs in association with suggestive family history (consanguinity and affected siblings), transfusion-dependent anemia from early childhood, inappropriately low reticulocyte counts, teardrop poikilocytes, and radiographic findings of symmetrical cortical thickening and diaphyseal widening of the long bones with metaphyseal sparing.

## Conclusions

We report the first documented co-inheritance of WAS and GHD in a child. This dual genetic diagnosis resulted in a blended phenotype of microthrombocytopenia, immunodeficiency, transfusion-dependent anemia, and early marrow fibrosis, features that could not be explained by either condition alone. The case highlights how rare disorders may interact to modify disease presentation, complicate diagnosis, and limit therapeutic choices, such as the contraindication of corticosteroids or aspirin. Hematopoietic stem cell transplantation was considered at least for the first condition (WAS), with doubtful benefit in the second. This case underscores the indispensable role of molecular diagnostics in clarifying atypical cytopenias, particularly in consanguineous families, and contributes to the global literature by expanding the phenotypic spectrum of inherited bone marrow failure syndromes.
